# Supplementary material for: Impact of a Surgical Approach on Endometrial Cancer Survival According to ESMO/ESGO Risk Classification: A Retrospective Multicenter Study in the Northern Italian Region
Source: Cancers (Basel). 2025 Jul 7;17(13):2261. doi: 10.3390/cancers17132261 (PMC12248672; doi:10.3390/cancers17132261)
Supplement: Supplementary file 1 [file cancers-17-02261-s001.zip › cancers-3679010-supplementary.pdf]

Supplementary Materials

Table S1. Adjuvant treatment administered in patients treated with laparotomy or laparoscopy stratified according to the ESMO-ESGO risk class. N-miss= missing data.

Risk class: LOW

	Laparoscopy (N=592)	Laparotomy (N=277)	Total (N=869)	p value
**Adjuvant treatment**				0.034
No	536 (90.8%)	238 (85.9%)	774 (89.3%)	
Yes	54 (9.2%)	39 (14.1%)	93 (10.7%)	
N-Miss	2	0	2	

Risk class: INTERMEDIATE

	Laparoscopy (N=122)	Laparotomy (N=144)	Total (N=266)	p value
**Adjuvant treatment**				0.529
No	44 (36.4%)	58 (40.3%)	102 (38.5%)	
Yes	77 (63.6%)	86 (59.7%)	163 (61.5%)	
N-Miss	1	0	1	

Risk class: INTERMEDIATE-HIGH

	Laparoscopy (N=192)	Laparotomy (N=95)	Total (N=287)	p value
**Adjuvant treatment**				0.023
No	56 (29.3%)	41 (43.6%)	97 (34.0%)	
Yes	135 (70.7%)	53 (56.4%)	188 (66.0%)	
N-Miss	1	1	2	

Risk class: HIGH

	Laparoscopy (N=243)	Laparotomy (N=451)	Total (N=694)	p value
**Adjuvant treatment**				0.401
No	62 (25.6%)	102 (22.8%)	164 (23.8%)	
Yes	180 (74.4%)	346 (77.2%)	526 (76.2%)	
N-Miss	1	3	4	

Risk class: ADVANCED/METASTATIC

	Laparoscopy (N=17)	Laparotomy (N=27)	Total (N=44)	p value
**Adjuvant treatment**				1.000
No	3 (17.6%)	4 (14.8%)	7 (15.9%)	
Yes	14 (82.4%)	23 (85.2%)	37 (84.1%)	

Histotype: ENDOMETRIOID

	Laparoscopy (N=1144)	Laparotomy (N=814)	Total (N=1958)	p value
**Adjuvant treatment**				< 0.001
No	748 (65.6%)	403 (49.6%)	1151 (59.0%)	
Yes	392 (34.4%)	409 (50.4%)	801 (41.0%)	
N-Miss	4	2	6	

Histotype: OTHERS THAN ENDOMETRIOID

	Laparoscopy (N=138)	Laparotomy (N=220)	Total (N=358)	p value
**Adjuvant treatment**				0.171
No	54 (39.4%)	70 (32.1%)	124 (34.9%)	
Yes	83 (60.6%)	148 (67.9%)	231 (65.1%)	
N-Miss	1	2	3	

Table S2. Cox multivariate analysis of principal clinical and therapeutic variables influencing overall survival in the total cohort of patients and in the sub-cohort of high risk and advanced/metastatic patients. Age was included as a covariate only in Model 1.

TOTAL COHORT

		Model 1			Model 2		
Variable		Hazard Ratio	CI.95	p-value	Hazard Ratio	CI.95	p-value
**Age**	**(continuous)**	-	-	-	1.06	[1.04;1.07]	<0.001
**Diabetes**	**No**	-	-	-	Ref		
	**Yes**	-	-	-	1.23	[0.87;1.73]	0.2435
**Hypertension**	**No**	-	-	-	Ref		
	**Yes**	-	-	-	1.07	[0.79;1.45]	0.6753
**ASA_score**	**I**	-	-	-	Ref		
	**II**	-	-	-	1.35	[0.49;3.72]	0.5672
	**III**	-	-	-	2.10	[0.75;5.85]	0.1559
	**IV**	-	-	-	3.62	[0.95;13.86]	0.0603
**Risk class**	**Low**	Ref			Ref		
	**Intermediate**	1.93	[1.13;3.31]	0.0167	1.49	[0.83;2.68]	0.1864
	**Intermediate-High**	1.93	[1.07;3.48]	0.0297	1.73	[0.89;3.36]	0.1039
	**High**	4.49	[2.99;6.76]	<0.001	3.77	[2.38;5.96]	<0.001

	**Advanced/Metastatic**	24.66	[13.73;44.31]	<0.001	18.34	[9.71;34.65]	<0.001
**Surgical Approach**	**LPT**	Ref			Ref		
	**LPS**	0.74	[0.56;0.99]	0.0435	0.76	[0.56;1.03]	0.0750
**Adjuvant therapy**	**No**	Ref			Ref		
	**Yes**	0.77	[0.57;1.03]	0.0781	1.04	[0.75;1.45]	0.8125

HIGH RISK COHORT							
		Model 1			Model 2		
Variable		Hazard Ratio	CI.95	p-value	Hazard Ratio	CI.95	p-value
**Age**	**(continuous)**	-	-	-	1.03	[1.01;1.06]	0.00457
**Diabetes**	**No**	-	-	-	Ref		
	**Yes**	-	-	-	1.18	[0.70;2.01]	0.53233
**Hypertension**	**No**	-	-	-	Ref		
	**Yes**	-	-	-	1.31	[0.84;2.04]	0.23145
**ASA_score**	**I**	-	-	-	Ref		
	**II**	-	-	-	1.68	[0.40;7.10]	0.48329
	**III**	-	-	-	1.97	[0.45;8.57]	0.36835
	**IV**	-	-	-	2.78	[0.37;21.17]	0.32233
**FIGO stage**	**I**	Ref			Ref		
	**II**	0.97	[0.49;1.92]	0.9393	0.85	[0.42;1.72]	0.65203
	**III**	1.76	[1.04;2.99]	0.0364	1.76	[1.03;2.99]	0.03768
**Histology**	**Endometrioid**	Ref			Ref		
	**Others**	1.88	[1.22;2.90]	0.0039	1.92	[1.23;2.97]	0.00372
**LVSI**	**No**	Ref			Ref		
	**Yes**	1.58	[1.01;2.46]	0.0460	1.55	[0.99;2.40]	0.05311
**Surgical Approach**	**LPT**	Ref			Ref		
	**LPS**	0.50	[0.31;0.82]	0.0058	0.48	[0.29;0.79]	0.00417
**Adjuvant therapy**	**No**	Ref			Ref		
	Yes	0.68	[0.42;1.09]	0.1109	0.86	[0.51;1.44]	0.56284

ADVANCED/METASTATIC COHORT							
		**Model 1**			**Model 2**		
Variable		Hazard Ratio	CI.95	p-value	Hazard Ratio	CI.95	p-value
**Age**	**(continuous)**	-	-	-	1.02	[0.92;1.13]	0.7343
**Diabetes**	**No**	-	-	-	Ref		
	**Yes**	-	-	-	0.11	[0.01;1.46]	0.0939
**Hypertension**	**No**	-	-	-	Ref		
	**Yes**	-	-	-	0.45	[0.09;2.25]	0.3311
**ASA_score**	**I**	-	-	-	Ref		
	**II**	-	-	-	0.27	[0.01;11.94]	0.4969
	**III**	-	-	-	0.97	[0.04;22.66]	0.9867
	**IV**	-	-	-	28.98	[0.27;3130.79]	0.1588
**Histology**	**Endometrioid**	Ref			Ref		
	**Others**	0.88	[0.26;3.02]	0.8429	2.65	[0.40;17.45]	0.3099
**LVSI**	**No**	Ref			Ref		
	**Yes**	1.24	[0.41;3.76]	0.7021	2.52	[0.56;11.29]	0.2258
**Surgical Approach**	**LPT**	Ref			Ref		
	**LPS**	0.36	[0.07;1.75]	0.2035	1.41	[0.18;11.03]	0.7443
**Adjuvant therapy**	**No**	Ref			Ref		
	**Yes**	0.06	[0.01;0.33]	0.0013	0.00	[0.00;0.08]	<0.001

Supplementary Figure 1

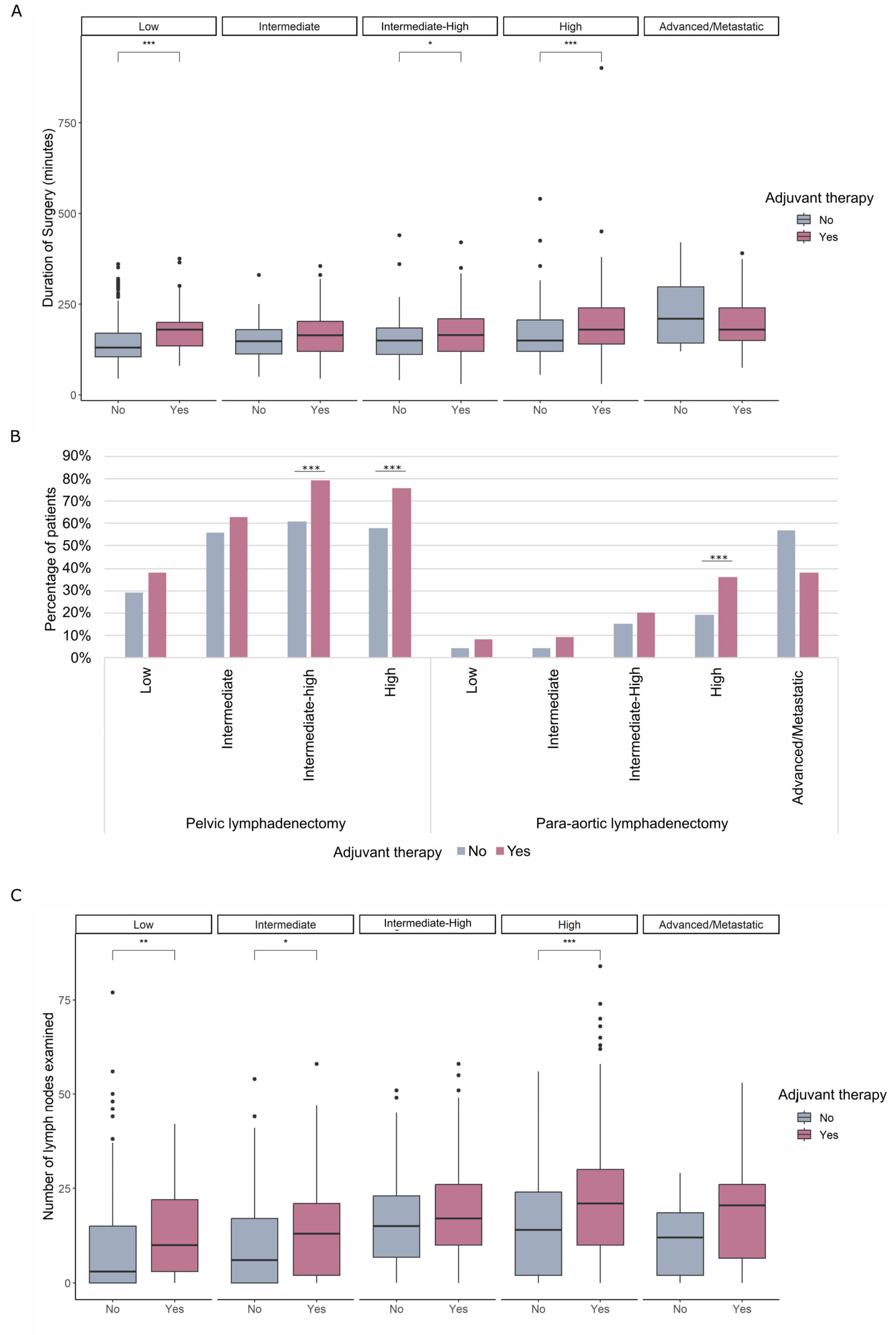

Figure S1. A. Boxplot comparing the duration of surgery in patients that subsequently needed or not adjuvant therapy, subdivided for risk class. B. Histograms representing the percentage of patients staged with pelvic or paraaortic lymph node dissection, subdivided by risk class and differentiated between those who received or not adjuvant therapy. C. Boxplot comparing the number of lymph-nodes examined in patients who received staging procedures and subsequently needed or not adjuvant therapy, subdivided for risk class.
